# Bending and twisting the embryonic heart: a computational model for c-looping based on realistic geometry

**DOI:** 10.3389/fphys.2014.00297

**Published:** 2014-08-12

**Authors:** Yunfei Shi, Jiang Yao, Jonathan M. Young, Judy A. Fee, Renato Perucchio, Larry A. Taber

**Affiliations:** ^1^Department of Biomedical Engineering, Washington UniversitySt. Louis, MO, USA; ^2^Dassault Systemes Simulia Corp.Providence, RI, USA; ^3^L-3 Applied TechnologiesSan Diego, CA, USA; ^4^Department of Mechanical Engineering, University of RochesterRochester, NY, USA

**Keywords:** biomechanics, morphogenesis, cardiac looping, chick embryo, finite-element modeling

## Abstract

The morphogenetic process of cardiac looping transforms the straight heart tube into a curved tube that resembles the shape of the future four-chambered heart. Although great progress has been made in identifying the molecular and genetic factors involved in looping, the physical mechanisms that drive this process have remained poorly understood. Recent work, however, has shed new light on this complicated problem. After briefly reviewing the current state of knowledge, we propose a relatively comprehensive hypothesis for the mechanics of the first phase of looping, termed c-looping, as the straight heart tube deforms into a c-shaped tube. According to this hypothesis, differential hypertrophic growth in the myocardium supplies the main forces that cause the heart tube to bend ventrally, while regional growth and cytoskeletal contraction in the omphalomesenteric veins (primitive atria) and compressive loads exerted by the splanchnopleuric membrane drive rightward torsion. A computational model based on realistic embryonic heart geometry is used to test the physical plausibility of this hypothesis. The behavior of the model is in reasonable agreement with available experimental data from control and perturbed embryos, offering support for our hypothesis. The results also suggest, however, that several other mechanisms contribute secondarily to normal looping, and we speculate that these mechanisms play backup roles when looping is perturbed. Finally, some outstanding questions are discussed for future study.

## 1. Introduction

Cardiac looping is a fundamental unsolved problem during early heart development. Looping represents the first major morphological sign of left-right asymmetry in the vertebrate embryo. In addition, looping abnormalities likely underlie some of the cardiac malformations that occur in as many as 1% of liveborn and 10% of stillborn human births (Harvey, 1998), and such defects may result in numerous spontaneous abortions during the first trimester (Srivastava and Olson, 1997; Ramsdell, 2005). During the last 30 years, most research on this problem has focused, with considerable success, on genetics and molecular signaling (Harvey, 2002), while interest in biophysical mechanisms waned. As a result, the physical processes that create the looped heart tube (HT) have remained poorly understood.

During the last decade, we have used a combination of experiments and computational modeling to explore the mechanics of the first phase of looping, called c-looping, as the initially straight HT bends and twists into a c-shaped tube normally directed toward the right side of the embryo (Patten, 1922; Männer, 2000). Employing an engineering approach, we have identified several of the forces that are involved in c-looping and have proposed hypotheses for how these forces are integrated to produce a looped heart (Voronov et al., 2004; Latacha et al., 2005; Taber, 2006; Taber et al., 2010; Shi et al., 2014). Computational models with simplified heart geometry have been instrumental in testing the physical plausibility of our hypotheses for bending or rotation alone, but a realistic model for the entire c-looping process has not yet been published. Models are important complements to laboratory experiments, as intuition can be misleading when trying to interpret the results of highly non-linear problems such as looping, which involves multiple intrinsic and extrinsic forces as well as dramatically changing 3-D geometry.

Here, we present the first relatively comprehensive computational model for the early HT and use it to explore a new hypothesis for the mechanics of c-looping. This model extends and integrates our previous models for looping, which simulated bending and torsion separately (Taber et al., 1995; Taber and Perucchio, 2000; Voronov et al., 2004; Ramasubramanian et al., 2006, 2008; Shi et al., 2014). The model is built on a foundation of experimental data and is based on the fundamental principles of soft tissue mechanics, including large deformation, growth, and active cytoskeletal contraction. Novel features include contact between the HT and splanchnopleuric membrane and realistic 3-D geometry reconstructed from images of an embryonic chick heart (acquired via optical coherence tomography, OCT). For the same set of physical parameters, the model captures reasonably well the morphology of the looping heart under both control and mechanically perturbed conditions. This study lays the foundation for future patient-specific models for cardiac morphogenesis.

## 2. Background

The problem of cardiac looping has a long and tortuous history. Since the pioneering study of Patten (1922) nearly a century ago, researchers have proposed numerous hypotheses for the mechanisms of c-looping, but few have survived the test of time. (See Taber, 2006 and Shi et al., 2014 for recent reviews.) Here, we briefly summarize our current thinking on this topic.

**Figure 1:**
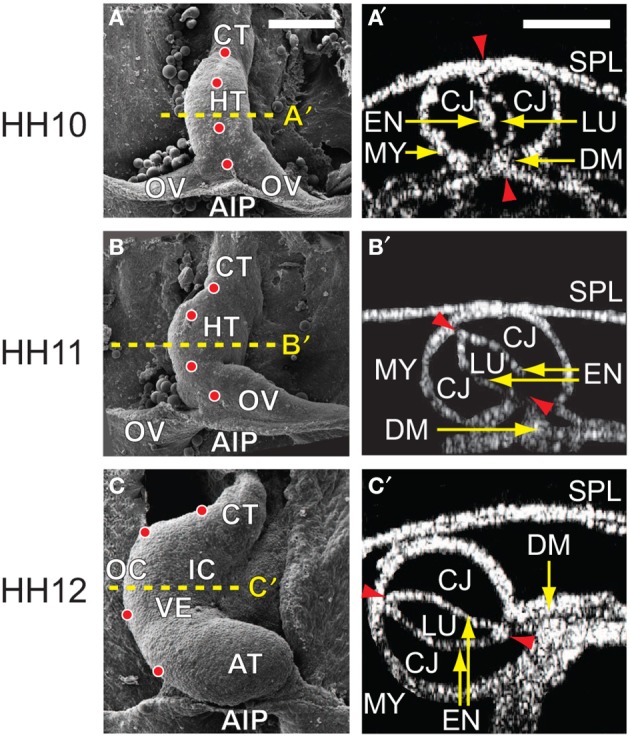
**Cardiac c-looping in chick embryo. (A–C)** SEM images of embryonic chick hearts during c-looping (ventral view). (Reprinted from Shi et al., 2014 with permission of ASME.) The originally straight heart tube (HT) at HH10 in **(A)** bends ventrally and rotates rightward, transforming into a c-shaped tube at HH12 in **(C)**. Note that artificial labels (red dots) along the ventral midline of the HT at HH10 move to the outer curvature of the HH12 heart. **(A′–C′)** Rotation of the HT is shown by the orientation of the elliptical lumen (red arrowheads) in OCT cross sections taken midway along the length of the HT [yellow dashed lines in **(A–C)**]. AIP, anterior intestinal portal; AT, atrium; CJ, cardiac jelly; CT, conotruncus; DM, dorsal mesocardium; EN, endocardium; IC, inner curvature; LU, lumen; MY, myocardium; OC, outer curvature; OV, omphalomesenteric vein; SPL, splanchnopleure; VE, ventricle. Scale bars: 200 μm. Yellow arrows indicate the annotated anatomic structures.

When it is first created in the chick embryo at stage HH10 of Hamburger and Hamilton (1951), the heart is a relatively straight tube consisting of an outer layer of myocardium, a middle layer of matrix called cardiac jelly (CJ), and an inner layer of endocardium (Figures 1A,A′). The HT is connected caudally to the omphalomesenteric veins (OVs), cranially to the conotruncus (outflow tract), and dorsally to the dorsal mesocardium (DM), which attaches the entire length of the HT to the foregut. In addition, the splanchnopleuric membrane (SPL) presses against the ventral side of the HT and wraps around the caudal sides of the OVs at the anterior intestinal portal (AIP) (Männer, 2000; Taber, 2006).

During c-looping, the HT undergoes a combination of ventral bending and rightward torsion (rotation) (Männer, 2000; Voronov et al., 2004; Taber, 2006). These deformations transform the ventral and dorsal surfaces of the initially straight HT into the convex outer curvature (OC) and concave inner curvature (IC), respectively, of the curved tube (Männer, 2000; Voronov et al., 2004) (Figures 1C,C′). In 3-D space, the looped heart acquires a helical shape (Bayraktar and Männer, 2014). During this process, the OVs gradually fuse to lengthen the HT, and the DM ruptures so only the ends of the HT remain connected to the embryonic foregut.

Studies suggest that the bending and torsional components of c-looping are driven by different sets of physical forces. While bending is caused mainly by forces generated within the HT, torsion is driven primarily by external loads (Butler, 1952; Voronov and Taber, 2002; Voronov et al., 2004; Latacha et al., 2005; Ramasubramanian et al., 2008). Proposed bending mechanisms include buckling as the HT outgrows the allotted distance between its ends (Patten, 1922; Bayraktar and Männer, 2014), dorsally constrained longitudinal stretching as CJ swells and inflates the HT (Manasek et al., 1984), differential hyperplastic growth of the myocardium (Stalsberg, 1969), active changes in myocardial cell shape (Manasek et al., 1972; Latacha et al., 2005; Auman et al., 2007), differential cytoskeletal contraction (Itasaki et al., 1991; Taber et al., 1995), and bending forces exerted on the HT by remnants of the DM after it ruptures (Taber et al., 1995).

Recently, we have shown that the bending component of c-looping can be attributed primarily to differential *hypertrophic* growth, although CJ swelling, active myocardial cell-shape change, and DM tension may play more minor roles. This idea is consistent with recent results of Soufan et al. (2006), who found that myocardial cells near the OC of the HT increase in volume during c-looping considerably more than cells near the IC. Prior to the study of Soufan et al. (2006), it was generally thought that the heart grows primarily by cellular hyperplasia before birth and hypertrophy after birth (Grossman, 1980), leading most researchers to rule out differential growth as a mechanism for looping when no clear mitotic patterns were found in the HT (Sissman, 1966; Stalsberg, 1969). In fact, this is one reason why we had previously supported a bending mechanism based on active changes in cell shape (Taber et al., 1995; Latacha et al., 2005; Ramasubramanian et al., 2006).

Interestingly, one of the forces involved in torsion of the HT was suggested more than 60 years ago by Butler (1952), who speculated that the left OV exerts a torque on the heart as it grows larger than the right OV. Recent studies support this hypothesis, as reducing the size of the left OV by dissection or inducing the right OV to grow larger than the left OV produces abnormal leftward looping (Voronov et al., 2004; Kidokoro et al., 2008). Other data suggest that proliferating cells on the left side of the DM normally provide a rightward push on the HT that determines looping direction (Linask et al., 2005), and cytoskeletal contraction in the OVs near the AIP also may be involved (Voronov et al., 2004). It is likely that multiple redundant mechanisms contribute to torsion of the heart, thus reducing the incidence of leftward looping, which is a major source of congenital heart defects (Ramsdell, 2005).

We have found, however, that the OVs normally cause only a relatively small amount of rightward torsion. Once looping direction is defined by asymmetric growth in the OVs or proliferative cellular forces in the DM, the SPL supplies a surface pressure that pushes the HT dorsally into its fully twisted position (Voronov and Taber, 2002; Voronov et al., 2004; Linask et al., 2005; Kidokoro et al., 2008).

**Figure 2:**
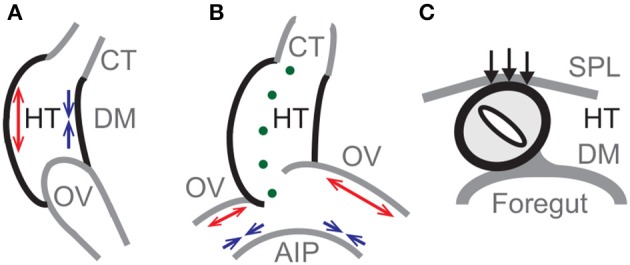
**Primary mechanisms in model for c-looping**. Schematic diagrams of looping heart tube (HT) in **(A)** lateral, **(B)** ventral, and **(C)** cross-sectional views. **(A)** Bending of the HT is driven mainly by differential hypertrophic myocardial growth. Longitudinal growth causes the ventral side to elongate (red arrows), while the dorsal side shortens (blue arrows) with the dorsal mesocardium (DM) located at the inner curvature. **(B)** Asymmetric growth (red arrows) on the cranial sides of the omphalomesenteric veins (OVs) causes a slight rightward twist of the HT, as cytoskeletal contraction on the caudal sides (blue arrows) enhances the OV forces exerted on the HT. (Green dots indicate the original midline of the HT.) **(C)** The splanchnopleuric membrane (SPL) pushes the HT dorsally (black arrows) into its fully twisted position. AIP, anterior intestinal portal; CT, conotruncus.

Based on these prior studies, the main forces that drive normal c-looping in our proposed model are differential growth in the HT and OVs, and pressure exerted by the SPL. Differential growth causes the HT to bend (Figure 2A) and the OVs to push against the HT, initiating a slight rightward twist (Figure 2B). Then, SPL pressure enhances the torsion as the HT continues to bend (Figure 2C). The model also includes growth of CJ, tension in the DM, active changes in myocardial cell shape, cytoskeletal contraction around the AIP, and elongation of the HT caused by OV fusion.^1^

## 3. Materials and methods

### 3.1. Preparation and culture of the embryonic heart

The methodology for preparation and culture of the chick embryo is adopted from Voronov and Taber (2002). Briefly, fertile white Leghorn chicken eggs (Sunrise Farms, Catskill, NY) were incubated in a humidified atmosphere at 38°C for 33–48 h to yield embryos at HH stages 10–12 (Hamburger and Hamilton, 1951). Embryos were extracted from the eggs using filter paper rings and rinsed in PBS. To eliminate surface tension artifacts, the sandwich structure of embryo and filter paper rings was covered with liquid culture media consisting of 89% Dulbecco's modified Eagle's medium (DMEM, Sigma), 10% chick serum (Sigma), and 1% antibiotics to a depth of approximately 5 mm. A stainless steel ring was placed on top of the paper rings to hold them in place. Culture dishes were then sealed in plastic bags filled with a humidified mixture of 95% O_2_ and 5% CO_2_, and put into an incubator for continued culture.

### 3.2. Measurement of HT rotation

Most experimental data used to inform and test our model come from previous studies. To this we add measurements of HT rotation as a function of time. Cardiac rotation was defined by the rotation of the lumen near the center of the HT. Images of the heart were acquired hourly from HH10- via OCT (Thorlabs, Newton, NJ) (Mesud Yelbuz et al., 2002), and 3-D tissue geometry was reconstructed using image analysis software (Volocity, PerkinElmer, Waltham, MA). Through image cropping, reslicing, and thresholding (ImageJ, NIH), the lumen in the chosen cross section was traced and fit to an ellipse. The rotation angle (α) is defined as the angle between the long axis of the fitted ellipse and the embryonic dorsal-ventral axis (see **Figure 5A**). All experimental measurements are presented as mean ± SD, and statistical analysis was performed using SigmaPlot software (Systat Software Inc., San Jose, CA).

### 3.3. Computational model geometry

A finite-element model was developed to simulate c-looping of the HT. The geometry was reconstructed from OCT images using Matlab (Mathworks, Natick, MA), PATRAN (MSC Software, Santa Ann, CA), and ABAQUS (SIMULIA, Providence, RI). Constitutive relations (material properties) and morphogenetic processes were defined via the ABAQUS user subroutine VUMAT (see below) with local material orientations determined using customized Matlab code. Simulations were carried out using the commercial finite-element code ABAQUS/Explicit, and some post-processing tasks were automated by execution of customized Python codes.

**Figure 3:**
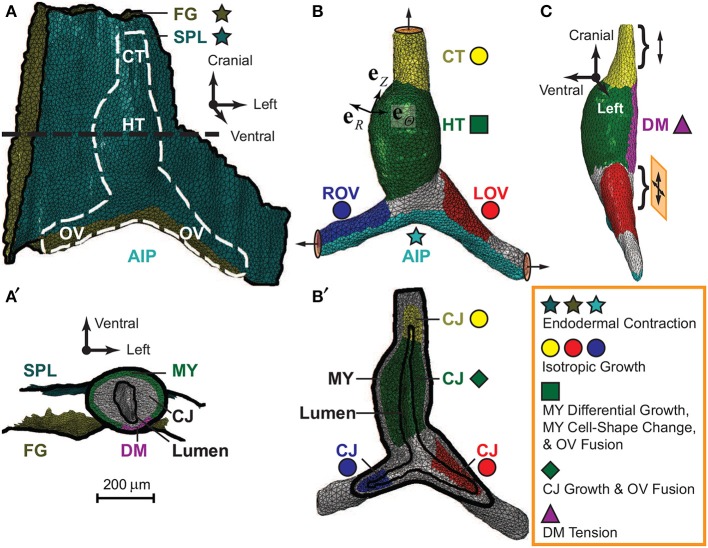
**Finite-element model for embryonic heart**. Model geometry in undeformed configuration was reconstructed from OCT images of a representative HH10- heart. **(A)** The complete model (ventral view) consists of the heart [white dashed line; see also **(B)**], which is sandwiched between the splanchnopleure (SPL) and the ventral wall of the foregut (FG). The caudal end of the SPL is attached to the caudal side of the omphalomesenteric veins (OVs) around the anterior intestinal portal (AIP), and the other membrane boundaries are fixed (black solid lines). **(B)** The heart model (ventral view) includes the heart tube (HT), conotruncus (CT), and the left and right OVs (LOV and ROV). The ends of the CT and OVs are constrained to move along the normal directions on their boundary surfaces (circular discs with arrows). **(C)** The dorsal mesocardium (DM), a narrow region along the dorsal side of the HT (side view), is free except near its cranial and caudal ends (braces), where it is anchored to the FG. The cranial end is on cranial-caudal oriented rollers (double-headed arrow), and the caudal end slides freely in the cranial-lateral plane (crossed double-headed arrows). **(A′)** Transverse cross section of the model [black dashed line in **(A)**]. Frictionless contact is enforced between the heart and the two external membranes (SPL and FG). In the HT, the myocardium (MY) wraps around the cardiac jelly (CJ), which encloses the lumen. Note that the long axis of the lumen lies approximately along the dorsal-ventral direction at HH10-. **(B′)** Frontal section of heart in **(B)**. The MY and CJ are divided into regions for prescribing morphogenetic processes (colored stars, circles, square, diamond, and triangle; see text for details) in local radial, circumferential, and longitudinal directions (**e**_*R*_, **e**_Θ_, **e**_*Z*_).

The initial geometry of our model was reconstructed from a stack of OCT images of an HH10- heart (Figure 3). Briefly, the SPL, foregut wall, myocardium, CJ, and lumen were segmented out from the images. (The endocardium was omitted in the current model.) Then, we extracted voxel surfaces at the interface of two entities and removed any voxel edges and vertices with sharp changes in local curvature, which could cause stress concentrations and convergence issues. The voxel surfaces were smoothed using Matlab to adjust the position of each vertex by its weighted average over the neighboring vertices. Then, the congruent and manifold triangular surface mesh was further smoothed and coarsened using the PATRAN Mesh-on-Mesh routine. Finally, the closed triangular surface mesh for the heart was converted to a solid tetrahedral mesh using ABAQUS/CAE.

Considering the geometric differences between the heart and surrounding membranes, our model includes two types of elements. The myocardium and CJ consist of 68,504 tetrahedral (C3D4) elements, and the SPL and foregut membranes consist of 10,231 triangular (M3D3) elements. The entire model contains 21,710 nodes. Testing with denser meshes showed that the chosen mesh is accurate enough for the present purposes.

### 3.4. Theory for modeling morphogenesis

The analysis of the model is based on a finite-element implementation of a biomechanical theory for large deformation and growth of soft tissue (Rodriguez et al., 1994). We have used this theory in previous work to simulate various morphogenetic processes. Here, we briefly discuss the basic idea; further details can be found in Shi et al. (2014).

Briefly, the total deformation of a psuedoelastic body is described by the deformation gradient tensor **F**, which maps material points from the reference configuration at HH10- to the deformed configuration at a later time. Growth and active contraction are simulated through the morphogenesis tensor M = F^*^^−1^ · **F**, which defines the local zero-stress configuration after the simulated processes. The elastic deformation gradient tensor F^*^ generates mechanical stress by enforcing geometric compatibility between material elements and accounting for the elastic response to applied loads. The Cauchy stress tensor σ is assumed to depend only on F^*^ through the constitutive relation

(1) $$\sigma =\frac{1}{J^{*}}F^{*}\cdot \frac{\partial W}{\partial E^{*}}\cdot F^{*\text{T}},$$

where *W*(E^*^) is the strain-energy density function, J^*^ = det F^*^ is the elastic volume ratio, and E^*^ = (F^*T^ · F^*^ − **I**) / 2 is the Lagrangian elastic strain tensor with **I** being the identity tensor and T denoting the transpose.

### 3.5. Material properties

Mechanical properties are based on the microindentation measurements of Zamir and Taber (2004). These authors characterized the myocardium and CJ of HH12 chick hearts as homogeneous isotropic materials using a strain-energy density function in the form

(2) $$W=\frac{A}{B}\left({\text{e}}^{B({\bar{I}}_{1}-3)}-1\right)+\frac{1}{D}\left(\frac{J^{*}{}^{2}-1}{2}-\ln J^{*}\right),$$

where *A* and *B* are material constants, and I_1_ = J^*^^−2/3^ tr (F^*T^ · F^*^) is a modified strain invariant. Since another study found no significant difference in end-diastolic stiffness between HH10 and HH12 hearts (Rémond, 2006), we assume that the material properties remain relatively unchanged throughout c-looping and take the above strain-energy density function for all tissues in our model.

For myocardium and CJ, we used the mean values of the mechanical parameters reported by Zamir and Taber (2004), i.e., *A*_MY_ = 13.0 Pa, *B*_MY_ = 0.57, *A*_CJ_ = 3.2 Pa, *B*_CJ_ = 0.39, and *D*_MY_ = D_CJ_ = 0.01. Although material properties for the SPL have not been measured, it is known that the SPL contracts and is under significant tension during looping (Voronov and Taber, 2002). Since contraction generally causes an increase in tissue stiffness, we chose *A*_SPL_ = 2*A*_MY_ = 26.0 Pa, *B*_SPL_ = B_MY_ = 0.57, *D*_SPL_ = D_MY_ = 0.01 for the SPL as well as the foregut. A sensitivity analysis shows that varying the value of *A*_SPL_ over a relatively large range has little effect on the overall deformation (e.g., rotation of the HT) or qualitative trends in strain and stress distributions (see Supplementary Figure S3B).

### 3.6. Boundary and contact conditions

The membrane representing the SPL wraps around the caudal side of the OVs and adheres to the OVs in the region of contact (Figures 3A,B). Otherwise, the SPL and foregut are taken as fixed along their margins (Figure 3A). The HT is sandwiched between these membranes, with frictionless contact enforced between them (Figure 3A′).

For the heart, the cranial end of the conotruncus and the lateral ends of the OVs are allowed to move only along the normal directions of their boundary surfaces (Figure 3B). Along the dorsal side of the HT, a narrow region is defined as the DM, which is free except at its cranial and caudal ends, where it is anchored to the foregut (Figure 3C). The cranial end of the DM is supported by cranial-caudal oriented rollers, while its caudal end is on 2-D rollers in the cranial-lateral plane to allow lateral rotations. These boundary conditions simulate growth and remodeling of surrounding tissues to accommodate growth of the heart. Finally, frictionless contact is imposed on the interior surface of the CJ to allow the lumen to collapse as CJ grows and swells (Figures 3A′,B′).

### 3.7. Local material orientations

To implement anisotropic morphogenetic processes for each anatomic region, the model geometry was used to define local radial (**e**_*R*_), circumferential (**e**_Θ_), and longitudinal (**e**_*Z*_) directions for each element (Figure 3B).

For the SPL and foregut, the radial direction (**e**_*R*_) is along the normal to the surface. Since the morphogenesis tensor is taken as isotropic in the local tangential plane of these membranes (see below), **e**_Θ_ and **e**_*Z*_ were arbitrarily chosen as any two orthogonal unit vectors within the element plane.

To define the normal direction in the heart, **e**_*R*_ was calculated as the weighted average of the exterior and interior surface normals by distances of the element centroid to these two surfaces. The longitudinal direction (**e**_*Z*_) is taken along the projection of 3-D curve connecting the transverse cross-sectional centroids of the HT or OVs onto the local myocardial plane. The circumferential direction (**e**_Θ_) is then the cross product of **e**_*Z*_ and **e**_*R*_.

### 3.8. Looping simulation

The baseline model for the looping HT under control conditions includes the essential morphogenetic processes and parameters contained in our recent model for bending of isolated hearts (Shi et al., 2014). Here, that model is extended to include the effects of the OVs and SPL, which drive torsion. This subsection provides details of the simulation procedure for the baseline model; model perturbations are described later. In general, the morphogenesis tensor is taken in the form

(3) $$M=M_{R}e_{R}e_{R}+M_{\Theta }e_{\Theta }e_{\Theta }+M_{Z}e_{Z}e_{Z},$$

with the components *M*_*I*_ (*I* = *R*, Θ, *Z*) being specified for each particular mechanism as functions of space and time relative to the reference configuration at HH10-.

***Morphogenesis in the HT.*** Our recent study of cultured HTs in isolation suggests that differential growth in the myocardium is the primary mechanism that drives the bending component of c-looping with CJ growth, DM tension, and active changes in myocardial cell shape contributing to a lesser degree (Shi et al., 2014). All of these mechanisms are included in the present model. Here, we briefly state the rationale for the chosen form of **M** for each mechanism as listed in Table 1.

**Table 1:** **Morphogenetic processes in baseline model**.

**Morphogenetic process**	**Morphogenesis tensor**	**Morphogenetic parameter**
Endodermal contraction	**M**_c_ = *M*^− 2^_c_ **e**_*R*_ **e**_*R*_ + *M*_c_ (**e**_Θ_ **e**_Θ_ + **e**_*Z*_ **e**_*Z*_)	*M*_c_ = 0.9 → 0.9 × 0.7^*^
		(SPL, Foregut, and AIP)
CJ growth	**M**_j_ = *M*_j_ (**e**_*R*_ **e**_*R*_ + **e**_Θ_ **e**_Θ_ + **e**_*Z*_ **e**_*Z*_)	*M*_j_ = 1.1 → 1.1 × 1.3 (HT)
DM tension	**M**_t_ = **e**_*R*_ **e**_*R*_ + **e**_Θ_ **e**_Θ_ + *M*_t_ **e**_*Z*_ **e**_*Z*_	*M*_t_ = 1.0 → 0.8 (DM)
Myocardial differential growth	**M**_g_ = *M*_g_ (**e**_*R*_ **e**_*R*_ + **e**_Θ_ **e**_Θ_) + *G*/*M*^2^_g_ **e**_*Z*_ **e**_*Z*_	*M*_g_ = 1.0 → 1.3 (HT)
	*G*(Θ) = det **M**_g_ = 2 Θ / π + 1^§^	
Myocardial cell-shape change	**M**_s_ = **e**_*R*_ **e**_*R*_ + *S* **e**_Θ_ **e**_Θ_ + *S*^− 1^ **e**_*Z*_ **e**_*Z*_	*M*_s_ = 1.0 → 1.3 (HT)
	*S*(Θ) = (*M*_s_ − 1) · (Θ / π − 1)^2^ + 1	
OV fusion^†^	**M**_f_ = **e**_*R*_ **e**_*R*_ + **e**_Θ_ **e**_Θ_ + *M*_f_ **e**_*Z*_ **e**_*Z*_	*M*_f_ = 1.0 → 1.5 (HT)
Growth in the OVs and CT^‡^	**M**_i_ = *M*_i_ (**e**_*R*_ **e**_*R*_ + **e**_Θ_ **e**_Θ_ + **e**_*Z*_ **e**_*Z*_)	*M*_i_ = 1.0 → 1.3 (CT),
		1.6 (Left OV), 1.2 (Right OV)

* Initial contraction *M*_c_ = 0.9 used as the initial condition at HH10-; additional contraction of 0.7 occurs during looping, and the total contraction is 0.9 × 0.7 = 0.63. Same notation also applies to other parameter values.

§ Θ ∈ [*0*, π] is the circumferential angle relative to the DM.

† Longitudinal growth in the HT for modeling OV fusion does not contribute to reported strains.

‡ CT = conotruncus.

The differential growth tensor **M**_g_ provides a spatial gradient in myocardial hypertrophic growth, which increases from the IC to the OC, consistent with the measurements of Soufan et al. (2006). The myocardial cell-shape tensor **M**_s_ was determined from SEM images of Manasek et al. (1972), who found that cells at the OC spread out while those near the IC elongate circumferentially during c-looping. Isotropic growth of CJ is given by **M**_j_, which was deduced from an estimate of myocardial stress in the looped heart (Zamir and Taber, 2004). Finally, the DM tension tensor **M**_t_ shortens the zero-stress length of the DM by an amount that yields myocardial stress and strain distributions in reasonable agreement with experimental data (Shi et al., 2014).

The spatial distributions of morphogenetic variables and parameters were extrapolated from our study of the isolated heart (Shi et al., 2014). However, differences in looping morphology between isolated and intact hearts required some minor adjustments. The most significant change is that in the present model, we do not include myocardial contraction, which is an adaptive response triggered by the removal of normal compressive loads exerted by the SPL (Nerurkar et al., 2006; Filas et al., 2011). In addition, the CJ growth parameter *M*_j_ increases from an initial value of 1.1 at HH10- to 1.43 at HH12 (see Table 1), consistent with the 1.3-fold increase suggested by the myocardial residual strain determined by Zamir and Taber (2004). Finally, the initial value *M*_j_ = 1.1 was chosen to yield the modest myocardial tension present at that stage (Shi et al., 2014).

To implement the morphogenetic functions in a piecewise continuous manner, the HT was further divided into 24 subregions, and the average values of the parameters were uniformly assigned for each subregion. This procedure reduced stress concentrations near boundaries between adjacent subregions.

***Endodermal contraction.*** Previous experiments have shown that the endoderm comprising the SPL and the caudal regions of the OVs at the AIP is in a state of relatively isotropic contraction during c-looping (Voronov and Taber, 2002; Voronov et al., 2004; Varner and Taber, 2012). We assume that endodermal contraction occurs isovolumetrically and take the contraction tensor in the form **M**_c_ = *M*^−2^_c_ **e**_*R*_ **e**_*R*_ + *M*_c_ (**e**_Θ_ **e**_Θ_ + **e**_*Z*_ **e**_*Z*_) with the constant volume constraint det **M**_c_ = 1 satisfied. The contraction parameter *M*_c_ in these regions was chosen to decrease (i.e., contractile strength increases) from 0.9 at HH10- to 0.63 at HH12 (see Table 1). These values were estimated from experimental data provided by Voronov et al. (2004) and Ramasubramanian et al. (2006).

***Growth in the OVs and Conotruncus.*** Recent results suggest that asymmetric growth in the OVs plays an important role in determining looping directionality (Voronov et al., 2004; Ramasubramanian et al., 2006, 2008; Kidokoro et al., 2008). Here, we used the limited data that are available to estimate growth in the OVs as well as in the conotruncus. We assume that growth along the cranial sides of OVs (both myocardium and CJ) is isotropic and take **M**_i_ = M_i_ (**e**_*R*_ **e**_*R*_ + **e**_Θ_ **e**_Θ_ + **e**_*Z*_ **e**_*Z*_), where *M*_i_ is the growth parameter (see Table 1). Since the left OV grows noticeably larger than the right OV during c-looping (Stalsberg and DeHaan, 1969; Voronov et al., 2004; Kidokoro et al., 2008), we chose *M*_i_ = 1.6 for the left OV and 1.2 for the right OV based on the morphogenetic strains between HH10- and HH11 measured by Ramasubramanian et al. (2006). Notably, the value of *M*_i_ has a relatively small effect on HT torsion (see Supplementary Figure S4). For the conotruncus, which bulges near the end of c-looping (Männer, 2000) (see also Figure 1C), we estimated *M*_i_ = 1.3 (i.e., det **M**_i_ = M^3^_i_ ≈ 2). This value is based on measurements of cardiomyocyte growth and proliferation provided in Soufan et al. (2006), who found little change in cell number but approximately a two-fold increase in cell size in the conotruncus from HH10- to HH12.

***Fusion of the OVs.*** During c-looping, the HT lengthens as new segments are added to its caudal end by fusion of the bilateral OVs (Voronov et al., 2004; Taber, 2006; Abu-Issa and Kirby, 2008; Kidokoro et al., 2008; Varner and Taber, 2012). The details of this fusion process are not included in the present model. For a first approximation, however, we specify global longitudinal growth of the HT through a morphogenesis (OV fusion) tensor in the form **M**_f_ = **e**_*R*_ **e**_*R*_ + **e**_Θ_ **e**_Θ_ + *M*_f_ **e**_*Z*_ **e**_*Z*_, where *M*_f_ is the OV fusion parameter (see Table 1). This growth is taken as uniform throughout the myocardium and CJ in the HT. The value *M*_f_ = 1.5 was estimated from the observed change in length of the HT during c-looping. It is important to note that, since OV fusion adds material only at the caudal end of the HT, this process does not contribute to morphogenetic strains measured by tracking tissue labels. For comparison with experimental data, therefore, we exclude this uniform growth component from our strain calculations given by the model.

***Total Morphogenesis Tensor.*** The total morphogenesis tensor for each region is the product of all the individual morphogenesis tensors (Shi et al., 2014). It is important to point out that while some of these tensors share similar mathematical expressions (e.g., **M**_j_ and **M**_i_, as well as **M**_t_ and **M**_f_), they represent fundamentally different mechanisms and function in different regions (see Figure 3). In our previous model for isolated hearts, morphogenetic processes were assumed to occur in sequential steps to demonstrate how each individual process affects the results (Shi et al., 2014). Here, we include only two steps—an initial step to reach the reference stage of HH10- and a looping step spanning the entire c-looping process from HH10- to HH12. The first step includes only the initial CJ growth and endodermal contraction; the second step includes everything else (see Table 1). All the morphogenetic parameters are assumed to change linearly over time, as suggested by time plots of measured myocardial strains in isolated hearts (Shi et al., 2014).

***Perturbations.*** To test the model, we used it to simulate looping under a variety of experimental perturbations (Voronov et al., 2004; Rémond et al., 2006; Kidokoro et al., 2008; Ramasubramanian et al., 2008). In each case, the parameter values are the same as those in the baseline model, unless stated otherwise. The perturbations include the following:

- To simulate the effects of contraction inhibitors (e.g., blebbistatin), contraction is turned off by setting *M*_c_ = 1 everywhere.
- To simulate inhibition of OV fusion, the uniform component of longitudinal growth is turned off in the HT by setting *M*_f_ = 1. Since it is unclear whether the DM ruptures in hearts with OV fusion blocked, we set the DM either free or on cranial-caudal oriented rollers along its length.
- To simulate mechanical perturbations involving dissection, the appropriate parts were removed from the model (e.g., SPL, conotruncus, OVs, or HT), and some boundary conditions were adjusted accordingly to eliminate rigid-body motions. Since contraction is not necessary for normal c-looping, we neglect the possible effects of contraction triggered by dissection (Nerurkar et al., 2006; Rémond et al., 2006; Filas et al., 2011).

### 3.9. Comparing numerical and experimental results

To compare the results yielded by our computational model with experimental data, we focus mainly on morphology, which is the most important criterion with the most abundant data. However, since multiple mechanisms can produce similar changes in shape (Stalsberg, 1970; Taber, 2006; Shi et al., 2014), it is essential also to consider other mechanical quantities such as stress and strain. To help visualize the deformation in the model (especially HT rotation), artificial labels are placed along the ventral midline of the HT or the OV junction (e.g., see **Figure 10**). Morphogenetic Lagrangian strains and HT rotation were quantified following the same procedures used in experiments. Since tissue stresses are difficult to measure accurately in intact embryos, we focus on the qualitative trends in stress, i.e., whether the tissue is under local tension or compression, rather than numerical values.

## 4. Results

In this section, results are first presented for the baseline model. Then, the ability of the model to predict the outcomes of various experimental perturbations is examined.

### 4.1. Baseline model for normal c-looping

**Figure 4:**
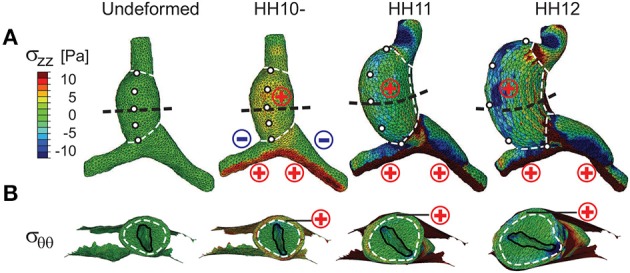
**Stress distributions in baseline model for c-looping. (A)** Longitudinal stress (σ_*zz*_) and **(B)** circumferential stress (σ_θθ_) are shown at three looping stages in ventral and transverse cross-sectional views [black dashed lines in **(A)**], respectively. To help visualize the deformation, white dashed lines divide the heart into regions, and artificial markers along the ventral midline of the undeformed heart tube (HT) indicate rotation. For comparison, “⊕” (red) and “øminus” (blue) signs denote regional myocardial tension and compression for the corresponding stress component, as previously revealed by microsurgery experiments (Voronov et al., 2004; Zamir and Taber, 2004; Shi et al., 2014). Note the rightward rotation of the HT as shown by the motion of the markers in **(A)** and the lumen orientation in **(B)**. Scale and legend are the same in **(A,B)**.

In general, the evolving morphology of the HT during c-looping given by the baseline model (Figure 4 and Supplementary Figure S2) agrees with experimental observations relatively well (compare with Figure 1). At the reference stage of HH10-, the initial CJ growth causes the HT to elongate slightly and the lumen to close slightly. At HH11, the HT is bent significantly with the DM located at the IC. Simultaneously, the HT twists (rotates) toward the right as it is pushed rightward by the rapidly growing left OV and compressed ventrally by the contracting SPL (see circular labels in Figure 4A). In addition, the length and circumference of the HT increase considerably. By the completion of c-looping at HH12, the OC and IC (original ventral and dorsal sides) of the HT have undergone considerable elongation and shortening, respectively, and the HT has fully rotated rightward with the lumen now oriented roughly along the embryonic lateral direction. Relative to HH11, the HT and left OV have grown significantly, and the conotruncus is bent rightward. All of these changes are consistent with normal c-looping *in ovo* (Männer, 2000; Taber, 2006) (see Figure 1).

**Figure 5:**
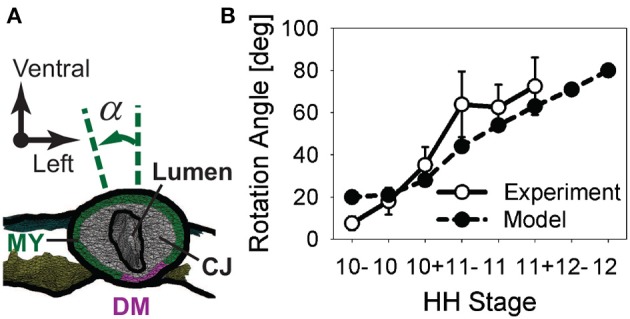
**Rotation of heart tube during c-looping. (A)** Rotation angle α is defined as the angle between the long axis of the elliptical lumen and the embryonic dorsal-ventral axis in a cross section located at the middle of the heart tube (CJ, cardiac jelly; DM, dorsal mesocardium; MY, myocardium). **(B)** Plot of rotation angle given by baseline model and experimental measurements as function of developmental stage.

To quantify torsion, we measured the rotation angle α of the HT in embryos cultured for 6 h from HH10- (Figure 5). The rotation angle increased as looping progressed, with most rotation occurring between HH10 (α = 18.0 ± 6.5 deg) and HH11 (α = 62.5 ± 10.8 deg; *n* = 5) (Figure 5B). The temporal plot of rotation angle given by our baseline model agrees with the experimental trend reasonably well (Figure 5B).

**Figure 6:**
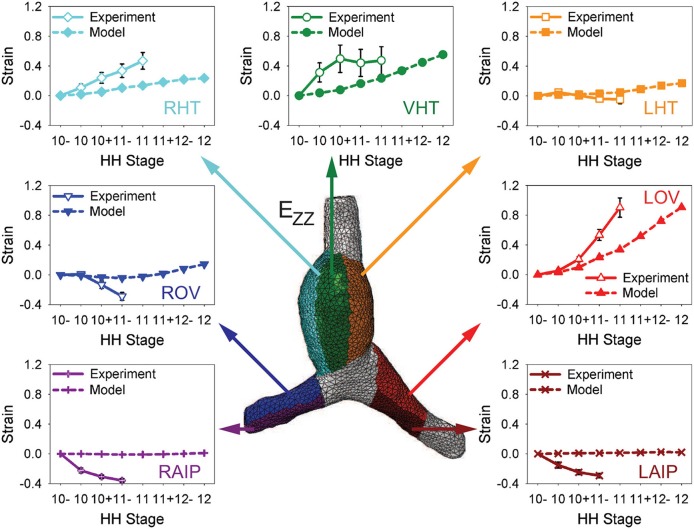
**Time history of regional longitudinal strains in baseline model**. Longitudinal strains (*E*_*ZZ*_) relative to HH10- in seven regions are compared with experimental data from Ramasubramanian et al. (2006). LHT/RHT/VHT, left/right/ventral sides of heart tube; LOV/ROV, cranial half of left/right omphalomesenteric veins; LAIP/RAIP, left/right sides of anterior intestinal portal.

**Figure 7:**
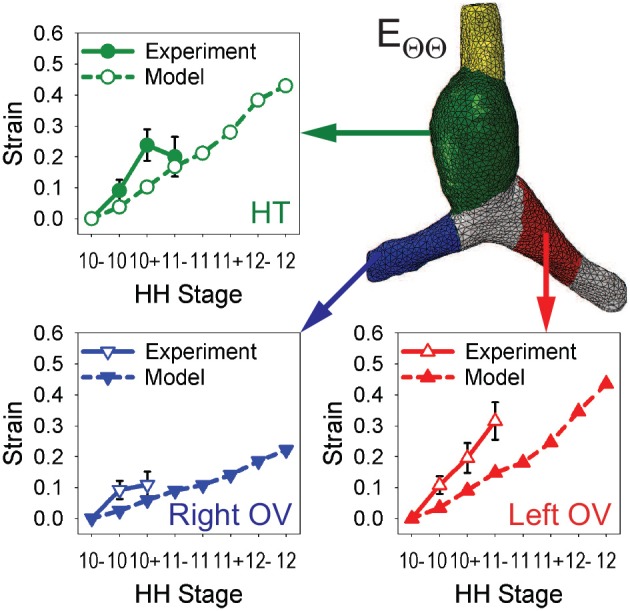
**Time history of regional circumferential strains in baseline model**. Average circumferential strains (*E*_ΘΘ_) relative to HH10- in three regions are compared with experimental data from Ramasubramanian et al. (2006). HT, heart tube; OV, omphalomesenteric vein.

To test the model further, myocardial strains relative to HH10- given by the model are compared with the experimental measurements of Ramasubramanian et al. (2006). Longitudinal strains (*E*_*ZZ*_) and circumferential strains (*E*_ΘΘ_) were averaged over seven regions and three regions, respectively, and plotted as functions of stage (Figures 6, 7). Overall, the general trends given by the model agree reasonably well with the data, although the strain magnitudes are somewhat smaller in the model. The discrepancy is most apparent for *E*_*ZZ*_ in the caudal parts of the OVs, where endodermal contraction causes longitudinal shortening, but the counterbalancing effects of CJ growth may have been overestimated.

In the HT, the longitudinal strain, which is closely associated with bending, increases on the original ventral and right sides while it changes relatively little on the left side (mainly due to compression by the SPL). Growth of the myocardium and CJ causes the average circumferential strain to increase in the HT and both OVs. On the cranial side of left OV, the longitudinal strain also increases considerably during looping, but the cranial side of right OV is initially compressed somewhat in the longitudinal direction by the faster growing left OV.

The trends in stress distributions given by the model also generally agree with those estimated experimentally (Voronov and Taber, 2002; Voronov et al., 2004; Zamir and Taber, 2004; Shi et al., 2014) (Figure 4; σ_*zz*_ and σ_θθ_ represent longitudinal and circumferential stresses, respectively). Due to the initial CJ growth, relatively uniform myocardial tension (σ_*zz*_, σ_θθ_ > 0) is present everywhere in the HT at HH10-. Endodermal contraction around the AIP generates longitudinal tension (σ_*zz*_ > 0) and compression (σ_*zz*_ < 0) on the caudal and cranial sides of OVs, respectively. These effects increase in the OVs as looping progresses. In the HT, however, the original myocardial tensions gradually decrease and even disappear in some regions. As discussed in Shi et al. (2014), much of this reduction in tension is caused by the myocardial growth that drives bending. Compression of the HT by the SPL is another contributing factor.

Taken together, our baseline model produces looping morphology, as well as strain and stress distributions, that are qualitatively consistent with available experimental data.

### 4.2. Effects of inhibiting contraction and vein fusion

Studies have shown that c-looping is relatively normal when non-muscle myosin II-based contraction is inhibited after looping begins at HH10 (Rémond, 2006; Rémond et al., 2006). Before HH10, however, blocking contraction prevents fusion of the OVs, and the heart does not loop. Rather, the OVs appear to be buckled (Supplementary Figure S1), and cardia bifida can occur (Rémond, 2006; Rémond et al., 2006; Varner and Taber, 2012). These effects also occur when OV fusion is blocked by dissection of endoderm at the center of the AIP (DeHaan, 1959; Nadal-Ginard and García, 1972; Kidokoro et al., 2008).

**Figure 8:**
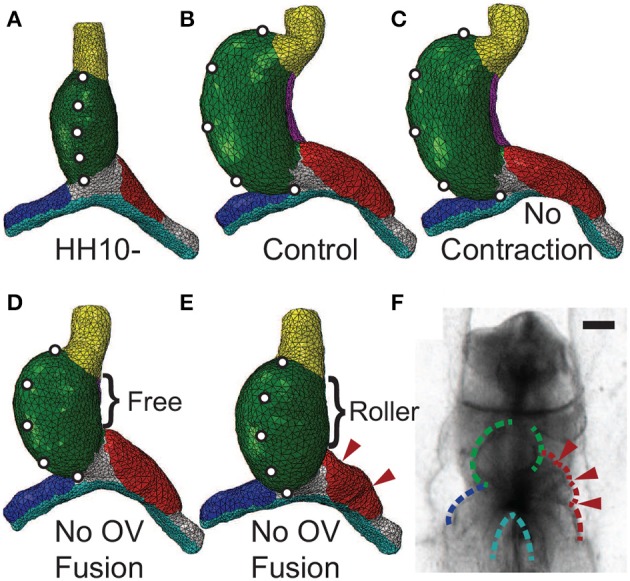
**Effects of inhibiting contraction or vein fusion. (A)** Straight heart tube (HT) at HH10-. To help visualize the deformation, artificial markers are placed along the ventral midline. **(B)** Baseline (control) model loops to the right at HH12. **(C)** Baseline model with cytoskeletal contraction turned off in the splanchnopleure, foregut, and the caudal sides of the omphalomesenteric veins (OVs) around the anterior intestinal portal. Consistent with experiments (Rémond et al., 2006), the heart loops relatively normally without contraction. **(D,E)** Baseline model with simulated OV fusion (uniform longitudinal growth in HT) turned off. Along the dorsal side of the HT, the dorsal mesocardium (DM) is either **(D)** ruptured (free) or **(E)** attached to the foregut (roller). Without OV fusion, the heart undergoes less bending and rotation than control **(D)**. With the DM further constrained **(E)**, relatively little rightward rotation occurs, and compression causes the cranial side of the left OV to buckle (arrowheads). **(F)** Buckled left OV in HH9 embryo exposed to 30 μM blebbistatin cultured for 20 h (see Supplementary Figure S1). Scale bar: 200 μm.

When contraction is turned off in our model (*M*_c_ = 1), looping morphology is relatively normal, although the diameter of the left OV is smaller than normal (Figures 8A–C). This decrease in vein diameter is caused by the drop in contraction-induced tension around the AIP that normally produces longitudinal compression and a corresponding increase in diameter on the cranial side of the vein.

When OV fusion is turned off (*M*_f_ = 1), the heart still loops but it bends and rotates less (Figure 8D). This behavior contradicts the experimental findings described above, and we reasoned that this inconsistency can be attributed to the DM not rupturing as it normally does when the HT bends. To simulate an intact DM, we added cranial-caudal oriented rollers along the full length of the DM. This added constraint eliminates almost all HT rotation (Figure 8E), and the left OV buckles on its cranial side (see arrowheads in Figure 8E). These results agree relatively well with experimental observations (Figure 8F; see also Supplementary Figure S1).

### 4.3. Effects of mechanical perturbations

Researchers have used various types of mechanical perturbations to explore the roles of external loads in the looping process (Nadal-Ginard and García, 1972; Voronov and Taber, 2002; Voronov et al., 2004; Taber, 2006; Kidokoro et al., 2008; Bayraktar and Männer, 2014). Most of these studies use dissection to disrupt the transmission of stress or to remove neighboring tissue. Similar to prior work with models of simplified geometry (Voronov et al., 2004; Ramasubramanian et al., 2008; Taber et al., 2010), we used results from these experimental studies to test our present model. Each experiment was simulated while keeping all model parameters unchanged.

**Figure 9:**
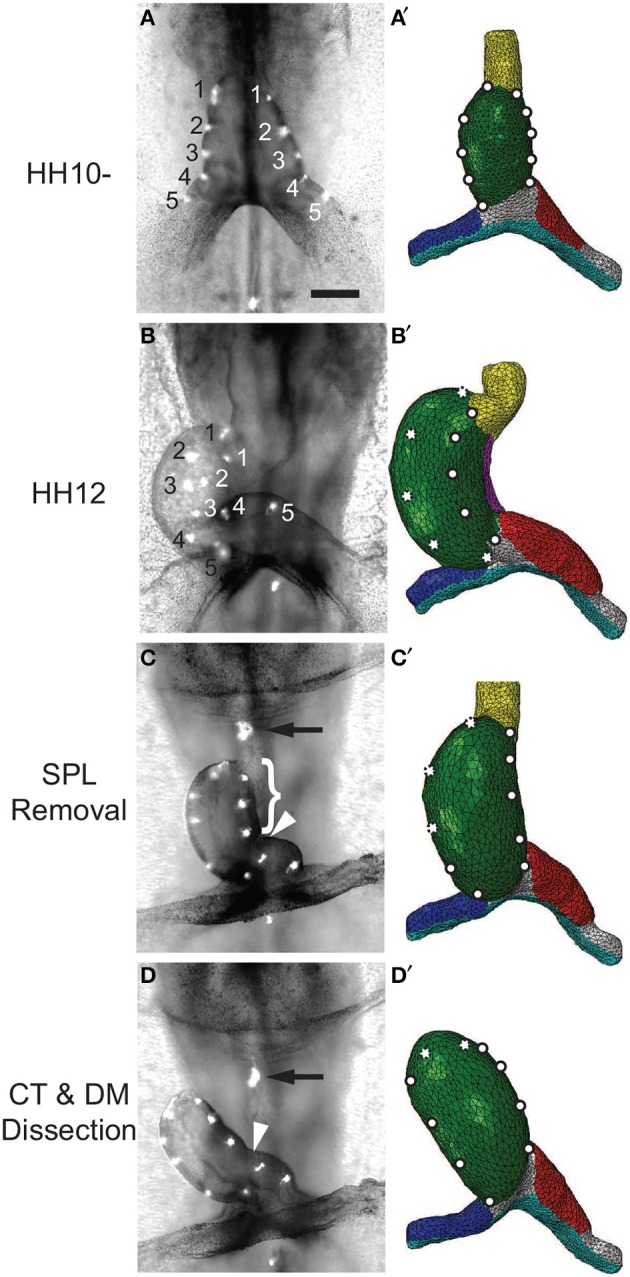
**Effects of removing external constraints on heart tube (HT). (A–D)** Bright-field images of experimental perturbations reprinted from Voronov et al. (2004) with permission of Elsevier. **(A′–D′)** Corresponding finite-element simulations. **(A,A′)** Straight HT at HH10-. To help visualize rotation, fluorescent labels were injected along the lateral sides of the HT in the experiment, and artificial labels are placed at similar locations in the model. **(B,B′)** Same heart at HH12 after 6 h of culture. As the heart rotates rightward, labels on the right (black numbers) and left (white numbers) sides of the HT move toward the dorsal and ventral sides, respectively. The model captures this phenomenon, as the labels originally on the right side now become invisible (dotted circles). **(C,C′)** Same heart at HH12 after removal of the splanchnopleure (SPL). In both the experiment and model, most rotation disappears as heart untwists, but heart remains bent slightly toward the right. **(D,D′)** Heart in **(C)** after transverse dissection of conotruncus (CT, black arrow) and longitudinal dissection of DM [brace in **(C)** shows length of the cut]. In both experiment and model, the HT tilts toward the right. In addition, the interventricular groove (white arrowhead) smooths out as the heart unbends. Scale bar: 200 μm.

Voronov et al. (2004) have shown that when the SPL is removed from an HH12 heart, the HT loses most of its rotation (Figures 9A–C; see also Supplementary Figure S3A). Then, after the conotruncus and DM are severed, the HT tilts to the right (Figure 9D). Our simulations for these dissections produced similar results (Figures 9A′–D′).

**Figure 10:**
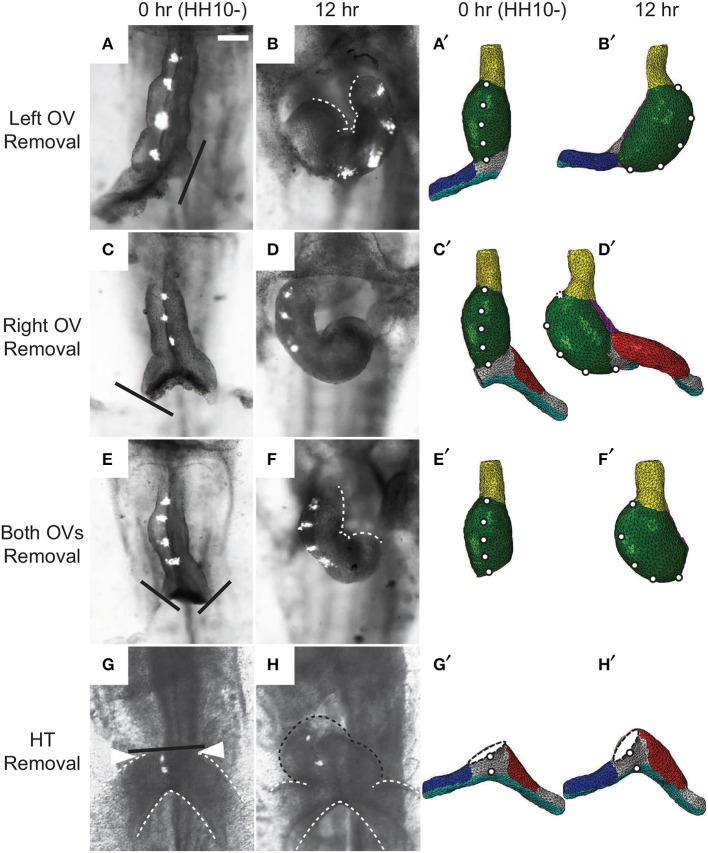
**Effects of removing omphalomesenteric veins (OVs) or heart tube (HT). (A–H)** Bright-field images of experimental perturbations reprinted from Ramasubramanian et al. (2008) **(A–F)** and Kidokoro et al. (2008) **(G,H)** with permissions of ASME and Wiley. **(A,C,E,G)** HH10- hearts with **(A)** the left OV, **(C)** the right OV, **(E)** both OVs, or **(G)** the HT removed. For access to the heart, the splanchnopleure was removed first. Black lines denote the cuts. To help visualize rotation, fluorescent labels were injected along the ventral midline of the heart. **(B,D,F,H)** The same hearts after 12 hr of culture. White dotted lines in **(B)** and **(F)** outline the inner curvature of the HT. **(A′–H′)** Corresponding finite-element simulations. The model predicts all of the final shapes reasonably well, including the leftward looping **(B,B′)**. Note that a portion of the HT [black dotted line in **(H)**] regrew above the interventricular grooves [arrowheads in **(G)**] in the experiment through OV fusion, which is not included in the model **(H′)**. Scale bar: 200 μm.

In other experiments, Ramasubramanian et al. (2008) removed the SPL and either one or both OVs. After 12 h of culture, the heart looped leftward when the left OV was removed and rightward when either the right OV or both OVs were removed (Figures 10A–F). These results suggest that looping direction can be determined by unbalanced lateral forces exerted by the OVs, and, without the counterbalancing effects of the dissected vein, the remaining vein pushes the HT to the opposite side. Our model reproduces all of these results reasonably well, including the left looping case, although there are some discrepancies in the morphology of the remaining vein (Figures 10A′–F′).

Ramasubramanian et al. (2008) attributed the observed HT rotation in the absence of the SPL and both OVs to a contractile response that occurs on the right side of the HT when the SPL is removed (Nerurkar et al., 2006; Ramasubramanian et al., 2008). The present model, which does not include this additional contraction, shows that initial geometric left-right asymmetry in conjunction with constraints imposed on the HT also can contribute to this torsion. At HH10-, the right side of the HT is slightly longer than the left side (Kidokoro et al., 2008) (see also Figures 3B,B′), causing the HT to bend slightly toward the right as it elongates while constrained by the DM at each end. This causes the caudal end of the HT to shift slightly leftward, which is consistent with the physical model results of Bayraktar and Männer (2014), and the continued caudal DM attachment to the foregut converts the bending into a rightward twist.

Finally, Kidokoro et al. (2008) removed the HT and found that the OVs continued to fuse and regrow a portion of the HT, which tilted rightward (Figures 10G,H). Although our model with the HT removed does not include vein fusion, it captures the morphology of the OVs relatively well (Figures 10G′,H′).

Taken together, these comparisons show that our model is able to predict the outcomes of various mechanical perturbations remarkably well without altering model parameters. Hence, these results support our integrated hypothesis for the physical mechanisms of c-looping.

## 5. Discussion

Cardiac looping has been one of the most studied and perplexing problems in morphogenesis. Although the genetic and molecular aspects of looping are now becoming clear, the mechanistic side of the story has remained poorly understood. Clearly, bending and twisting the HT requires mechanical forces, and researchers have proposed and tested a number of hypotheses for the biomechanics of looping. However, none of these hypotheses are consistent with all available data.

In the past decade, we have examined some of these ideas in considerable detail from a biomechanical engineering perspective. Using a combination of computational modeling and experiments, we have proposed a new hypothesis for the physical mechanisms that cause the HT to bend and twist during c-looping. According to our hypothesis, bending is driven primarily by forces generated within the HT, while twisting is caused mainly by external loads. Specifically, we have found that ventral bending is likely caused by differential hypertrophic growth in the myocardium (Shi et al., 2014), whereas torsion is driven by a combination of unbalanced forces exerted by the OVs on the HT that determine looping directionality and a compressive load applied by the SPL that enhances torsion (Figure 2) (Voronov and Taber, 2002; Voronov et al., 2004; Ramasubramanian et al., 2008; Taber et al., 2010).

Separate models for the bending and torsional components of c-looping have demonstrated the feasibility of these hypotheses (Taber et al., 1995; Voronov et al., 2004; Latacha et al., 2005; Nerurkar et al., 2006; Ramasubramanian et al., 2006, 2008; Shi et al., 2014). However, those previous models are based on simplified geometries and do not include coupling between bending and torsion. Hence, one purpose of the present study is to examine these hypotheses using a more comprehensive model that includes both bending and torsion as well as realistic geometry.

Our general strategy was to first develop a baseline model for normal c-looping. Model parameters for the HT are based on previous estimates (Shi et al., 2014), although some were modified slightly to account for differences in morphology between isolated and intact hearts. Forces generated within the HT include CJ swelling, tension in the DM, differential myocardial growth, and active changes in myocardial cell shape (Figures 3B,C,B′). External forces include those exerted on the HT by the SPL and OVs. Vein forces are generated by growth and cytoskeletal contraction on their cranial and caudal sides, respectively, (Figures 3B,B′). Our model yields relatively good agreement with normal heart morphology, as well as the qualitative trends in stress, strain, and HT rotation (Figures 4–7). Because of significant variability in the normal shape of the heart during looping (von Dassow and Davidson, 2007), we reason that matching spatiotemporal trends is a more realistic approach than insisting on precise quantitative agreement between numerical and experimental results.

Perhaps more importantly, for the same parameter values, the model predicts reasonably well the effects of various mechanical perturbations of the looping process (Figures 8–10). According to our hypothesis, looping direction is determined by a left-right difference in lateral forces exerted by the OVs, which push against the HT. Normally, the left OV is larger and exerts more pushing force than the right OV, causing the HT to twist slightly rightward (Voronov et al., 2004; Taber, 2006; Kidokoro et al., 2008; Ramasubramanian et al., 2008; Taber et al., 2010). However, if the left OV is removed or if growth of the right OV is enhanced, the right vein pushes the heart leftward, resulting in left looping (Kidokoro et al., 2008; Ramasubramanian et al., 2008). Experiments also have shown that the SPL causes most of the remaining torsion, since little torsion occurs when the SPL is removed before HH12 (Voronov et al., 2004; Nerurkar et al., 2006). Our model captures the results from these perturbation experiments quite well (Figures 9, 10 and Supplementary Figures S3A, S4E). The behavior of the model also is consistent with the finding that cytoskeletal contraction is not necessary for looping (Rémond, 2006; Rémond et al., 2006) (Figure 8C and Supplementary Figure S3A).

Recently, Bayraktar and Männer (2014) used a physical model to show that a growing tube constrained within the pericardial cavity (formed by the SPL and foregut) buckles into a helical shape consistent with the shape of the c-looped HT. This mechanism for bending extends the original idea of Patten (1922) that the HT buckles as it grows longer within a confined space, but the authors acknowledge that this idea seems inconsistent with bending of isolated hearts (Bayraktar and Männer, 2014). These authors also show that looping direction can be determined by a small initial offset in the lateral position of the caudal end of the HT. The present model does not rule out these mechanisms as possible contributing factors in c-looping, and, in fact, torsion of the HT in both models is caused primarily by constraints imposed by the SPL.

We suggest that recent experimental data support the hypothesis that differential hypertrophic growth drives the bending component of c-looping. For example, our model predicts a decrease in longitudinal stress near the OC as looping progresses (Figure 4A), in agreement with experimental results (Shi et al., 2014). In contrast, this stress would be expected to increase as the tube bends in the physical model of Bayraktar and Männer (2014).

Bayraktar and Männer (2014) also speculate that in embryos with cardia bifida, oppositely directed offsets between the left and right hemi-hearts may explain why the convex OCs of these hemi-hearts face the embryonic midline (Nadal-Ginard and García, 1972). Our model suggests an alternative explanation, i.e., that the left OV pushes the left hemi-heart rightward and the right OV pushes the right hemi-heart leftward (Figures 10A′–D′).

Taken together, the present results support the physical plausibility of our integrated hypothesis for c-looping. However, we must note that our model does not include all possible mechanisms that may be involved in this process. For example, it does not explicitly include OV fusion, which is simulated by global longitudinal growth of the HT (see Figures 3, 8D,E). The forces involved in the fusion process may affect looping (DeHaan, 1967; Nadal-Ginard and García, 1972; Rémond et al., 2006; Kidokoro et al., 2008; Varner and Taber, 2012). Our model also does not include left-right differences in growth of the DM as found by Linask et al. (2005). These authors speculated that a higher proliferation rate on the left side of the DM pushes the HT rightward and vice versa. This could be one of the redundant mechanisms that help make looping a relatively robust developmental process.

Finally, our model does not include mechanical feedback, which is a subject of increasing interest in developmental biology (Beloussov, 1998, 2008, 2012; Nerurkar et al., 2006; Ramasubramanian and Taber, 2008; Taber, 2008, 2009; Pouille et al., 2009; Bayly et al., 2013). For example, we have found that when the SPL is removed at HH10, little torsion occurs prior to HH11. Upon further culture, however, the data suggest that an abnormal contraction on the right side of the HT restores normal torsion by HH12 (Nerurkar et al., 2006; Ramasubramanian et al., 2008). This contraction appears to be a reaction to changes in the normal loads exerted on the heart (Nerurkar et al., 2006; Filas et al., 2011).

In the future, similar models could be used to determine the biophysical links between various genetic mutations and observed looping defects. This would entail conducting parameter studies by changing one model parameter at a time and comparing predicted and observed morphologies. However, it is important to keep in mind that multiple mechanisms can produce similar morphologies (Stalsberg, 1970; Taber, 2006; Shi et al., 2014). To hone in on precise mechanisms, it may be necessary to further develop techniques for measuring stress and strain fields non-invasively (Velduis and Brodland, 1999; Blanchard et al., 2009; Grashoff et al., 2010; Campàs et al., 2014).

In conclusion, our model is consistent with a variety of available data and supports the hypothesis that the bending component of c-looping is driven primarily by differential hypertrophic growth, while torsion is caused by forces exerted by the OVs and SPL. Looping directionality is dictated by unbalanced forces exerted by the OVs, with normal rightward looping caused by the left vein pushing with more force than the right vein. While these may be the primary mechanical forces involved in c-looping, it is likely that other forces also are involved, thus minimizing abnormalities in this crucial morphogenetic process.

## Author contributions

Yunfei Shi, Jiang Yao, and Larry A. Taber created the computational model, carried out the simulations, and drafted the manuscript. Jiang Yao, Jonathan M. Young, and Renato Perucchio created the model geometry. Yunfei Shi and Judy A. Fee conducted the experiments. Jonathan M. Young, Judy A. Fee, and Renato Perucchio revised the manuscript critically.

### Conflict of interest statement

The authors declare that the research was conducted in the absence of any commercial or financial relationships that could be construed as a potential conflict of interest.

## Footnotes

^1^In this paper, the term *contraction* refers to cytoskeletal contraction based on non-muscle myosin II, not the sarcomeric contraction that causes the heartbeat. Studies have shown that the embryonic chick heart loops normally in the absence of a heartbeat (Manasek and Monroe, 1972; Rémond et al., 2006; Shi et al., 2014).
