# Supplementary material for: Bending and twisting the embryonic heart: a computational model for c-looping based on realistic geometry
Source: Front Physiol. 2014 Aug 12;5:297. doi: 10.3389/fphys.2014.00297 (PMC4129494; doi:10.3389/fphys.2014.00297)
Supplement: Supplementary file 1 [file Presentation1.PDF]

Frontiers in Physiology

Research Article
July 31, 2014

Supplementary Material: Bending and Twisting the Embryonic Heart: A Computational Model for C-Looping Based on Realistic Geometry

Yunfei Shi [1,†], Jiang Yao [2,†], Jonathan M Young [3], Judy A Fee [1], Renato Perucchio [4], and Larry A Taber [1,*]

[1] *Dept. of Biomedical Engineering, Washington University, St. Louis, MO, USA*
[2] *Dassault Systemes Simulia Corp., Providence, RI, USA*
[3] *L-3 Applied Technologies, San Diego, CA, USA*
[4] *Dept. of Mechanical Engineering, University of Rochester, Rochester, NY, USA*

Correspondence*:
Larry A Taber
Dept. of Biomedical Engineering, Washington University, St. Louis, MO, 63130, USA, lat@wustl.edu
[†] *Yunfei Shi and Jiang Yao are co-first authors.*

Mechanotransduction and Development of Cardiovascular Form and Function.

1 SUPPLEMENTARY METHODS

To examine the effects of inhibiting contraction on vein fusion and looping prior to HH10-, embryos ($n > 5$) were harvested at HH9+ (approximately 30 hr of incubation) and cultured in medium containing 30 µM (-)blebbistatin (Sigma-Aldrich, St. Louis, MO). A stock solution of 30 mM (-)blebbistatin in dimethylsulfoxide was diluted 1:1000 in the culture medium to give the final concentration of 30 µM. For controls, the same amount of the solvent dimethylsulfoxide was added. To prevent photoinactivation of blebbistatin during culture and manipulation, exposure to light was limited and aluminum foil was used to cover the dishes whenever possible. The medium was replaced with fresh medium and blebbistatin after exposure to light during microscopy. Effectiveness of drug treatments was verified by diminished but not abolished heartbeat and reduced tissue tension.

2 SUPPLEMENTARY RESULTS

2.1 CONTRACTION INHIBITION

If myosin II-based contraction is inhibited before HH9, the OVs fail to fuse to create the HT [1, 2]. Once looping starts at HH10, however, OV fusion and looping appear normal when contraction is blocked [1, 3]. To test our model with OV fusion turned off, we acquired new experimental results for contraction inhibition prior to HH10-.

In control embryos, the OVs fused normally as indicated by the descending AIP and increasing length of the HT (Figures S1A–C). After 20 hr of culture, embryos reached HH13+ with c-looping completed

(Figure S1C). In blebbistatin-treated embryos, the OVs stopped fusing and the AIP did not descend (Figures S1D–F). The HT looped slightly, but its length remained relatively constant. Moreover, both OVs enlarged significantly, and the cranial side of left OV appeared to buckle (see arrowheads in Figure S1F).

2.2 ADDITIONAL COMPUTATIONAL RESULTS

Additional computational results include:

- Deformation of the HT given by the baseline model is shown in ventral and lateral views (Figure S2).
- Time histories of rotation angle obtained from two representative perturbation simulations (looping with contraction inhibition or removal of the SPL) are compared with experimental data for normal c-looping (Figure S3A).
- Sensitivity analysis of baseline model (see below) was conducted to investigate how torsion of the HT is affected by changing material properties of the SPL (Figure S3B) and growth of the OVs (Figure S4).

2.3 SENSITIVITY ANALYSIS OF BASELINE MODEL

To investigate further how torsion of the HT is affected by SPL and OVs forces, we varied the parameter values for the material modulus of the SPL (A_{SPL}) and growth of the OVs (M_{i}).

When the dimensionless SPL modulus $A' = A_{\mathrm{SPL}}/A_{\mathrm{MY}}$ increases from 0.5 to 4 ($A' = 2$ for baseline model), the time history plot of rotation angle changes little (Figure S3B).

When symmetric growth ($M_{\mathrm{i}} = 1$, 1.2, and 1.4) is specified for both OVs, the HT always twists rightward with similar rotation angles (Figures S4A–D). This is because in the undeformed model, the left OV is somewhat larger than the right OV, introducing an initial left-right asymmetry. Thus, growing at the same rate, the left OV remains larger than the right OV.

To simulate looping caused by chemically induced overgrowth in the right OV [4], we specify $M_{\mathrm{i}} = 1$ for the left OV and 1.8 for the right OV. The results show that at HH12, the initial geometric bias is overcome by the faster growing right OV, and the HT twists leftward (Figure S4E).

Taken together, these results are generally consistent with previous experimental data.

REFERENCES

[1] Rémond MC, Fee JA, Elson EL, Taber LA. Myosin-based contraction is not necessary for cardiac c-looping in the chick embryo. *Anat. Embryol. (Berl).* **211** (2006) 443–54. doi:10.1007/s00429-006-0094-0.

[2] Varner VD, Taber LA. Not just inductive: A crucial mechanical role for the endoderm during heart tube assembly. *Development* **139** (2012) 1680–90. doi:10.1242/dev.073486.

[3] Rémond MC. *Mechanics of the actomyosin cytoskeleton during looping of the embryonic chick heart.*. D.Sc. Thesis, Washington University in St. Louis (2006).

[4] Kidokoro H, Okabe M, Tamura K. Time-lapse analysis reveals local asymmetrical changes in c-looping heart tube. *Dev. Dyn.* **237** (2008) 3545–56. doi:10.1002/dvdy.21662.

FIGURES

SUPPLEMENTARY FIGURE S1: Effects of inhibiting contraction before looping begins. (A–C) HH9+ embryo cultured in control conditions for **(A)** 0 hr, **(B)** 7 hr, and **(C)** 20 hr. Note descending anterior intestinal portal (AIP; asterisk denotes the first pair of somites) as omphalomesenteric veins (OVs) fuse to form the heart tube (HT). **(D–F)** HH9+ embryo cultured in 30 μM (-)blebbistatin for **(D)** 0 hr, **(E)** 7 hr, and **(F)** 20 hr. AIP did not descend as OVs did not fuse. Instead, the OVs enlarged abnormally, and buckles (arrowheads) appeared along the cranial side of left OV. Scale bar: 200 μm.

SUPPLEMENTARY FIGURE S2: Results of deformed heart tube (HT) from baseline model for normal c-looping. Deformed HT is shown in ventral (upper panels) and lateral (lower panels) views. Arrows indicate the direction of lateral views. To help visualize rotation of the HT, artificial labels are placed along the ventral midline of the HT. Note that labels are invisible in left side view due to the rightward rotation of the HT.

SUPPLEMENTARY FIGURE S3: Effects of splanchnopleuric membrane (SPL) on torsion of heart tube (HT). (A) Rotation angle of HT given by the model (dashed lines) compared with experimental measurements (solid line). Torsion of the HT is not affected significantly by inhibition of cytoskeletal contraction (red line), whereas it is hindered when the SPL is removed from the model (blue line). **(B)** Increasing the dimensionless modulus for the SPL relative to the myocardium (A') has little effect on torsion of the HT.

SUPPLEMENTARY FIGURE S4: Effects of growth in omphalomesenteric veins (OVs) on torsion of heart tube (HT). Deformed model at **(A)** HH10- and **(B–E)** HH12. **(B–D)** When the same amount of growth is assigned for both OVs (M_i = 1 **(B)**, 1.2 **(C)**, and 1.4 **(D)**), the HT always twists rightward with similar rotation. Note that the sizes of both veins increase with the growth parameter M_i. **(E)** Leftward looping occurs when the right OV exceeds the left OV in size (M_i = 1 in left OV and 1.8 in right OV). These results suggest that looping directionality can be determined by unbalanced forces exerted by the OVs.

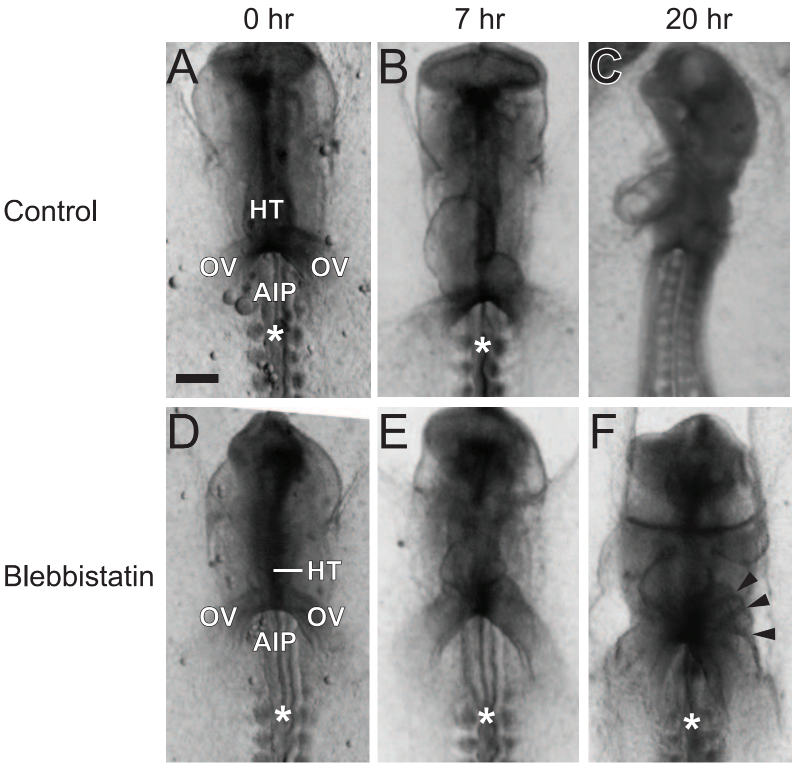

SUPPLEMENTARY FIGURE S 1. Effects of inhibiting contraction before looping begins.

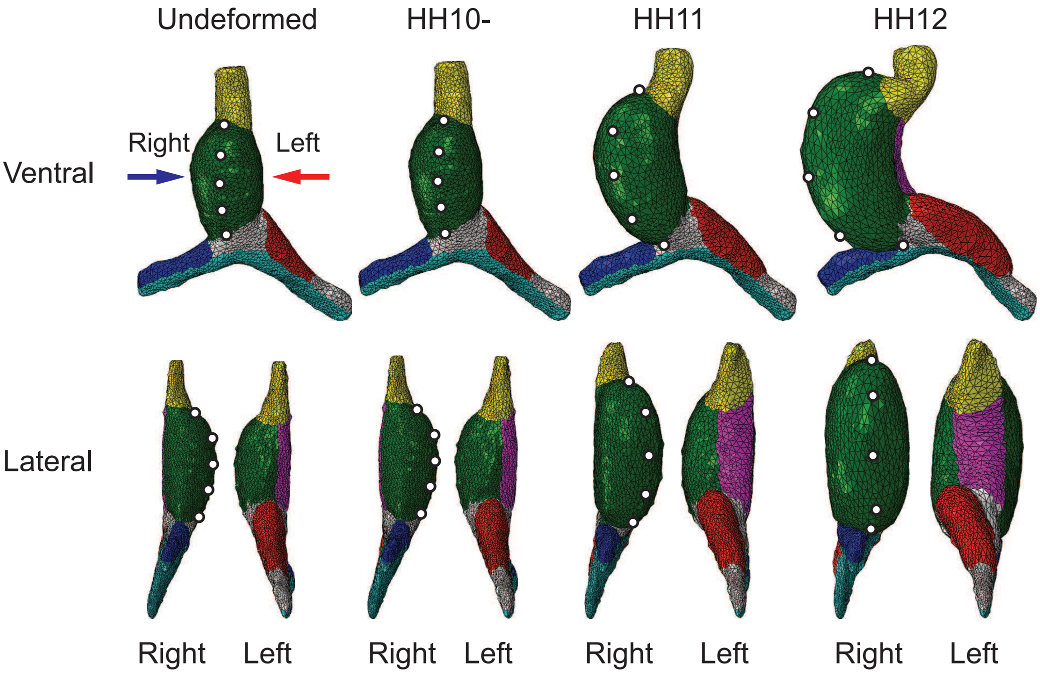

SUPPLEMENTARY FIGURE S 2. Results of deformed heart tube from baseline model for normal c-looping.

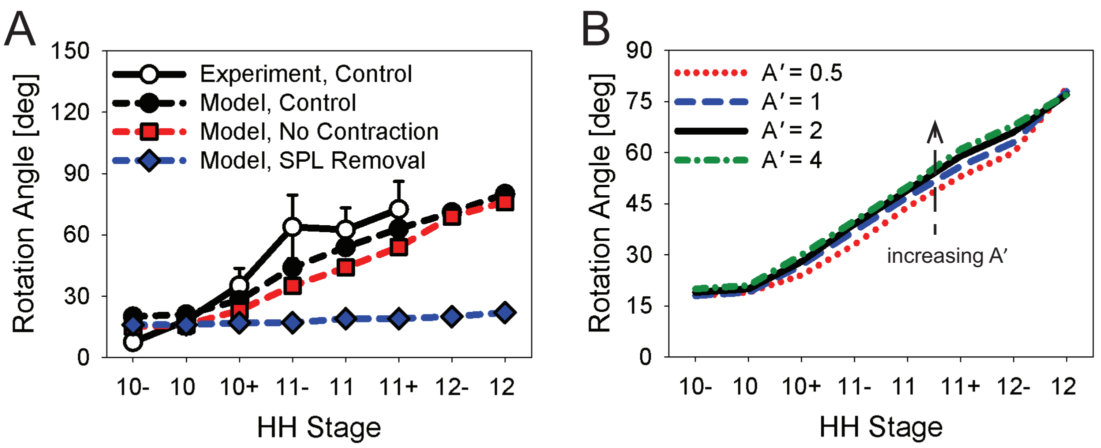

SUPPLEMENTARY FIGURE S 3. Effects of splanchnopleure on torsion of heart tube.

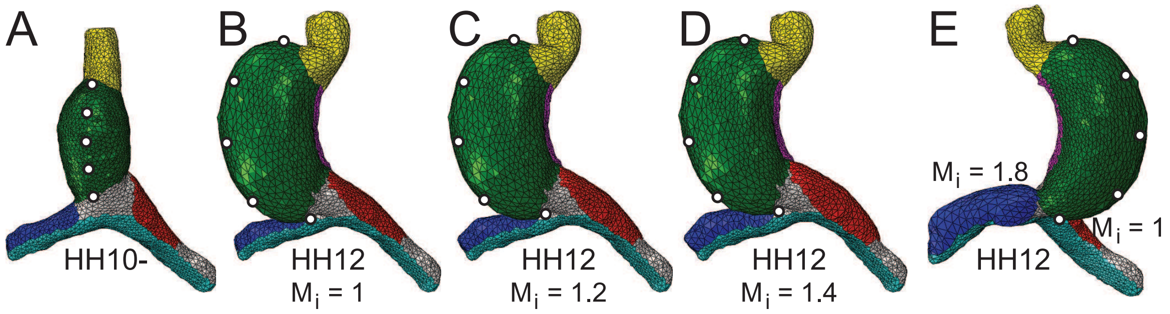

SUPPLEMENTARY FIGURE S 4. Effects of growth in omphalomesenteric veins on torsion of heart tube.
